# First-year real-world experience of intravitreal brolucizumab injection for refractory neovascular age-related macular degeneration

**DOI:** 10.1007/s10384-024-01134-7

**Published:** 2024-11-05

**Authors:** Jeong Hyun Lee, Joo Young Shin, Jeeyun Ahn

**Affiliations:** Department of Ophthalmology, Seoul National University College of Medicine, Seoul Metropolitan Government-Seoul National University Boramae Medical Center, Seoul, Korea

**Keywords:** Neovascular age-related macular degeneration, Brolucizumab, Intravitreal injection

## Abstract

**Purpose:**

To investigate the first-year real-world anatomical and functional outcomes of intravitreal brolucizumab injection in eyes with refractory neovascular age-related macular degeneration (nAMD).

**Study design:**

Retrospective observational study.

**Methods:**

nAMD patients who showed poor response to previous anti-vascular endothelial growth factor (VEGF) agents were switched to brolucizumab. Functional and anatomical outcomes were evaluated at initial treatment of nAMD, after treatment with other anti-VEGF agents and after switching and treating with brolucizumab for 1 year. Safety profile was also evaluated after brolucizumab injection. Best-corrected visual acuity (BCVA), central foveal thickness (CFT), subfoveal choroidal thickness (SFCT), and the presence of fluid in different compartments (intraretinal fluid [IRF], subretinal fluid [SRF], pigment epithelial detachment [PED]) were assessed at each time point.

**Results:**

A total of 40 eyes of 40 patients were included in the study. BCVA remained unchanged throughout treatment (*p* > 0.05). CFT did not change after treatment with other anti-VEGF agents (*p* = 0.588) but decreased after switching to brolucizumab (*p* < 0.001). SFCT decreased after treatment with other anti-VEGF agents (*p* = 0.025) but not after switching to brolucizumab (*p* = 0.236). Presence of SRF (*p* = 0.001) and PED (*p* = 0.001) decreased significantly after switching to brolucizumab, despite their persistence with prior treatments using other anti-VEGF agents. However, IRF persisted even after switching to brolucizumab (*p* = 0.745). Intraocular inflammation (IOI)-related adverse events were reported in 3 eyes (7.14%).

**Conclusion:**

Analysis of first-year real-world outcomes after switching to brolucizumab in nAMD patients refractory to other anti-VEGF agents showed improved anatomic outcomes, limited functional improvement and low incidence of IOI-related adverse events.

## Introduction

Age-related macular degeneration (AMD) is a chronic, progressive macular disease causing central visual loss [1, 2]. Although anti-vascular endothelial growth factor (VEGF) therapy has decreased legal blindness and visual impairment, AMD remains the third leading cause of severe vision loss worldwide [1–3]. By 2040, the number of people with AMD is expected to be nearly 300 million, posing a major public health problem at substantial socioeconomic cost [4].

Monthly visits for intravitreal injection and costly anti-VEGF drugs place high burden on both patients and caregivers. To reduce this burden, investigators have made efforts to develop new drugs that have higher efficacy and longer durability. Ranibizumab (Lucentis, Genentech /Roche), approved by the Food and Drug Administration (FDA) in 2006, was administered monthly up to 2 years in clinical trials [5]. Subsequently, in 2011, aflibercept (Eylea, Regeneron) was developed to extend the treatment interval up to 2 months [6, 7]. Recently, brolucizumab (Beovu, Novartis) was approved for treatment of neovascular AMD (nAMD) by the FDA in 2019, based on the HAWK and HARRIER studies, in which brolucizumab demonstrated non-inferiority to aflibercept with better anatomical outcomes and extended injection intervals [8]. Brolucizumab is a low molecular weight, single-chain antibody fragment that targets all forms of VEGF-A with high affinity [9]. However, intraocular inflammation (IOI)-related adverse events including retinal vasculitis with or without occlusion have been reported [10].

Outcomes with anti-VEGF agents in the real world fall short of those achieved in clinical trials which are strictly designed to gain approval from regulatory agencies due to lower patient compliance leading to less frequent injections [11–13]. Real-world short-term (1–3 months) outcomes of brolucizumab are reported in previous studies [14–16]. In current study, we aimed to evaluate first-year real-world anatomical and functional outcomes of intravitreal brolucizumab injections in eyes with refractory nAMD.

## Subjects and methods

### Participants

This was a retrospective, observational study performed with approval from the institutional review board of Seoul Metropolitan Government-Seoul National University (SMG-SNU) Boramae Medical Center (No. 30-2022-62), and in accordance with the Declaration of Helsinki. Medical records of nAMD patients who were switched to brolucizumab due to poor responsiveness to previous anti-VEGF agents were reviewed. Patients who were switched between September 2021 and November 2022 and regularly injected for at least 1 year were included in the study. Initial treatment for all nAMD patients consisted of three-monthly loading doses of anti-VEGF agent followed by pro re nata (PRN) treatment. During the course of PRN treatment, refractory nAMD was defined as persistent intraretinal fluid (IRF) or subretinal fluid (SRF) despite continued treatment with two consecutive doses of the same anti-VEGF agent. The exclusion criteria were: (1) presence of macular diseases other than nAMD; (2) history of uveitis; (3) significant media opacity affecting optical coherence tomography (OCT) quality; (4) IOI-related AEs as a form of vitritis or retinal vasculitis after brolucizumab injection.

### Treatment protocol and OCT acquisition

OCT examinations were performed using spectral-domain OCT (Spectralis; Heidelberg Engineering). Macular volume scans consisting of 31 horizontal high-speed B-scans covering 30°x 25° centered on the foveola were acquired at each visit. Brolucizumab was switched after at least 4 weeks for bevacizumab/ranibizumab and after 8 weeks for aflibercept. Intravitreal injections were performed in a clean room as an outpatient procedure on the day of the patients’ visit to the hospital for OCT examination. After pupil dilation, topical anesthesia, and sterile draping, 6 mg (0.05 ml) of brolucizumab was injected using a single-use prefilled syringe 3.5 mm posterior to the limbus. Patients were followed up monthly after brolucizumab injection for OCT examination. Patients received brolucizumab on a PRN regimen and the minimum injection interval was 2 months.

### Data collection and analysis

Demographic characteristics, history of previous anti-VEGF agent injections, choroidal neovascularization (CNV) subtype, functional/anatomical outcome, and AEs were collected. Functional outcomes were evaluated with best-corrected visual acuity (BCVA) and anatomical outcomes were evaluated with central foveal thickness (CFT), subfoveal choroidal thickness (SFCT) and presence of SRF, IRF, and pigment epithelial detachment (PED). Functional and anatomical outcomes were analyzed at initial treatment of nAMD, after treatment with other anti-VEGF agents (at initial brolucizumab injection) and after switching and treating with brolucizumab for 1 year.

### Statistical analysis

Continuous variables are expressed as mean and standard deviation and the categorical variables are expressed as absolute frequency and percentage. Normal distribution of examined variables were verified by Kolmogorov-Smirnov test. Paired comparison of continuous variables between each time point were analyzed using the Wilcoxon signed-rank test. Pearson’s chi-squared test or Fisher’s exact test was used to analyze presence of change of categorical variables between each time point. Statistical analyses were performed using IBM SPSS statistics ver. 27.0 (IBM Corp). P value < 0.05 was considered statistically significant. 

**Fig. 1:**
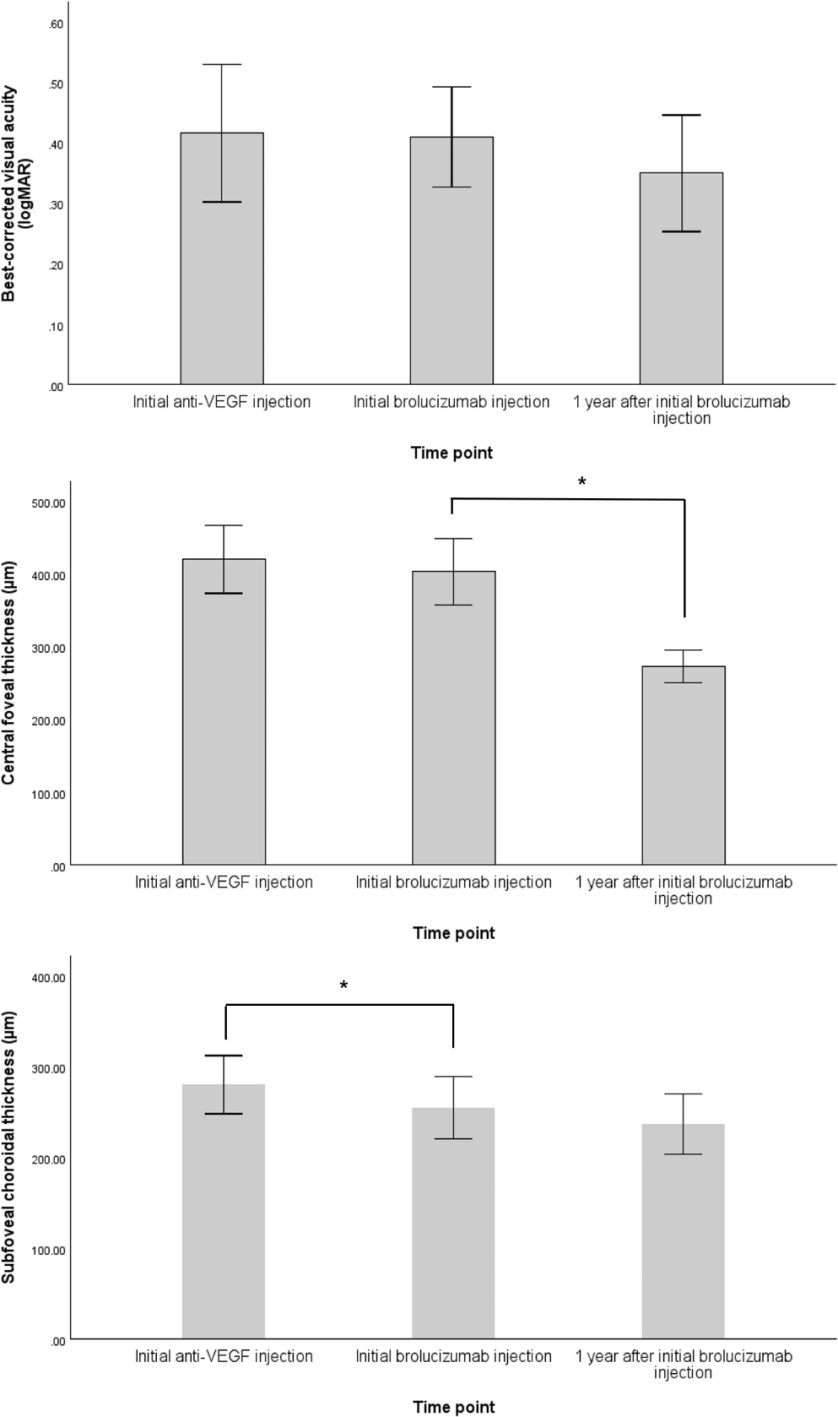
Best-corrected visual acuity, central foveal thickness, and subfoveal choroidal thickness at three time points *=significant change during the period

## Results

### Baseline characteristics

A total of 40 eyes of 40 patients were included in the study. Patients’ mean age was 74.08 ± 8.85 years and the majority were men (70.0%). Prior to treatment with brolucizumab, patients had received an average of 14.18 ± 9.16 injections over a period of 30.10 ± 29.05 months, with injections administered every 1.46 ± 0.60 months (0.67–2.88). On average, patients had been treated with 1.87 ± 0.73 different types of anti-VEGF agents. The patient with the least frequent injections (injection every 2.88 months) experienced recurrence three months after the initial loading injections of aflibercept. Most patients (45%) had received 2 types of anti-VEGF agents, while 20% had received 3 types. In becoming refractory to prior two consecutive anti-VEGF agents, 2 patients showed increase of SRF, and 1 patient showed slight decrease and remnant SRF. The rest of the patients (*n* = 37) showed similar amounts of SRF as evaluated by OCT. After switching to brolucizumab, patients received 6.03 ± 1.91 injections (range 3 to 7) of brolucizumab over a period of 12.38 ± 0.91 months. Patient baseline demographics are summarized in Table 1.

**Table 1:** Baseline demographics

	Total 40 eyes
Age (years)	74.08 ± 8.85
Male (n [%])/Female (n [%])	28 (70) / 12 (30)
BCVA (logMAR)	0.41 ± 0.33
CFT (µm)	419.97 ± 140.33
SFCT (µm)	280.05 ± 95.99
CNV type, n (%)	
Type 1	31 (77.50)
Type 2	9 (22.50)
Type 3	0
Fluid localization, n (%)	
IRF	5 (21.43)
SRF	40 (100)
PED	40 (100)
Previous treatment history	
Type of drug agent	1.87 ± 0.73
Number of injections	14.18 ± 9.16
Treatment period (month)	30.10 ± 29.05

**Table 2:** Functional and anatomical change over the period of three time points

	Initial anti-VEGF injection	Initial brolucizumab injection	1 year after initial brolucizumab injection	*P* ^a^	*p* ^b^
BCVA (logMAR)	0.41 ± 0.33	0.41 ± 0.25	0.35 ± 0.28	0.611^*^	0.224^*^
CFT (µm)	419.97 ± 140.33	402.81 ± 136.94	272.27 ± 67.50	0.588^*^	<0.001^*^
SFCT (µm)	280.05 ± 95.99	254.70 ± 102.52	236.57 ± 100.29	0.025^*^	0.236^*^
Lesion					
IRF, n (%)	5 (12.5)	7 (17.5)	5 (12.5)	0.531^†^	0.531^†^
SRF, n (%)	40 (100)	40 (100)	15 (37.5)		0.001^†^
PED, n (%)	40 (100)	40 (100)	30 (75.00)		0.001^†^

### Functional and anatomical outcomes

**Fig. 2:**
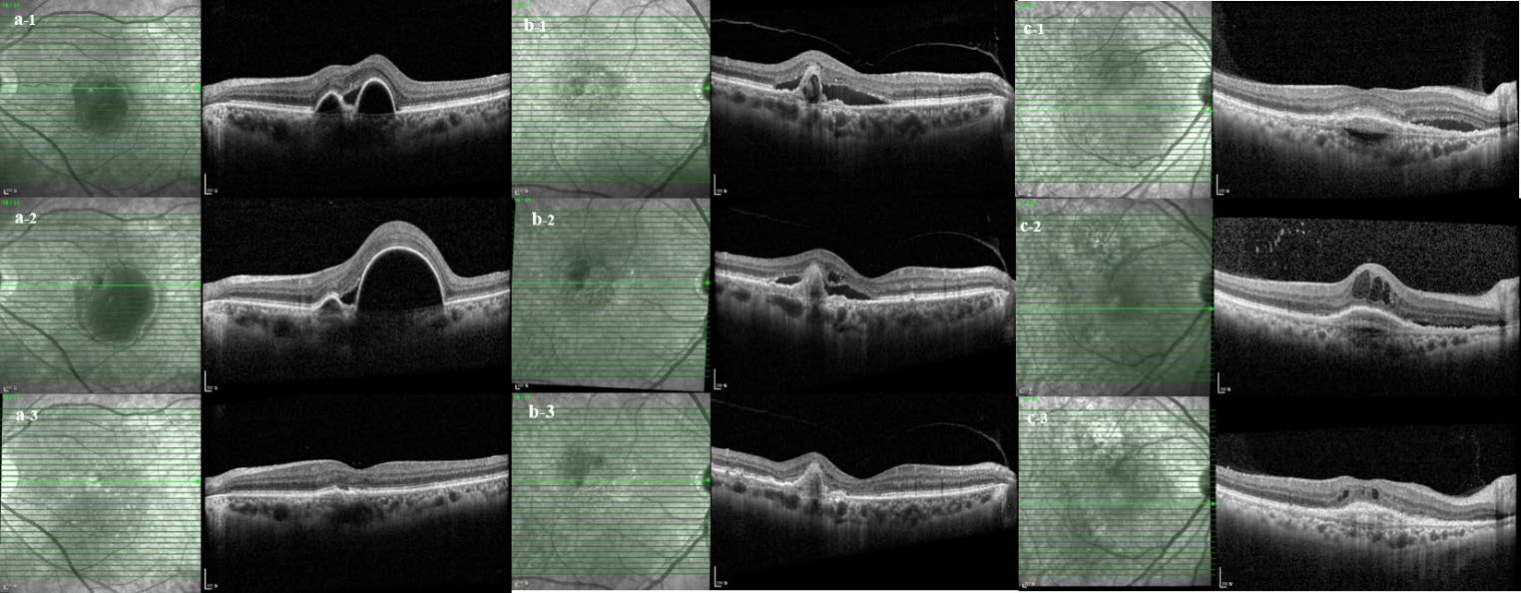
Optical coherence tomography images of three representative patients (**a**, **b**, **c**) at the time of nAMD diagnosis (**a-1**, **b-1**, **c-1**), on the day of the initial brolucizumab injection (**a-2**, **b-2**, **c-2**), and after one year of brolucizumab treatment (**a-3**, **b-3**, **c-3**). Patient a exhibited regression of both pigment epithelial detachment (PED) and subretinal fluid (SRF). Patient b demonstrated persistent PED, while patient c showed persistent PED and intraretinal fluid (IRF).

BCVA remained unchanged after treatment with other anti-VEGF agents (initial anti-VEGF injection vs. initial brolucizumab injection, 0.41 ± 0.33 logMAR vs. 0.41 ± 0.25 logMAR, *p* = 0.611) and no significant improvement was observed despite switching to brolucizumab (initial brolucizumab injection vs. 1 year after initial brolucizumab injection, 0.41 ± 0.25 logMAR vs. 0.35 ± 0.28 logMAR, *p* = 0.224). CMT did not change after treatment with other anti-VEGF agents (419.97 ± 140.33 μm vs. 402.81 ± 136.94 μm [*p* = 0.588]), but decreased significantly after switching to brolucizumab (402.81 ± 136.94 μm vs. 272.27 ± 67.50 [*p* < 0.001]). SFCT significantly decreased after treatment with other anti-VEGF agents (280.05 ± 95.99 μm vs. 254.70 ± 102.52 μm [*p* = 0.025]) but not after switching to brolucizumab (254.70 ± 102.52 μm vs. 236.57 ± 100.29 μm [*p* = 0.236]). Presence of SRF (*p* = 0.001) and PED (*p* = 0.001) decreased significantly after switching to brolucizumab, whereas the presence of IRF did not change significantly (*p* = 0.531). All the 40 eyes showed presence of SRF and PED on the day of the first brolucizumab injection and complete resolution was found in 25 eyes (62.5%) for SRF and 10 eyes (25%) for PED after 1 year. Of the 30 eyes with persistent PED, PED height decreased in 35 eyes (87.5%) and persisted in 5 eyes (12.5%). None showed increase in PED height. OCT images of three representative patients are shown in Fig. 2.

### IOI-related adverse events

Among patients switched to brolucizumab, 3 patients were reported to have IOI (1 iridocyclitis, 1 vitritis, 1 retinal vasculitis). Iridocyclitis was diagnosed 2 weeks after the initial brolucizumab injection. Vitritis was diagnosed 2 weeks after the second brolucizumab injection. Both iridocyclitis and vitritis were diagnosed during routine follow-up exam. The patient with vasculitis came in earlier than the appointment day due to floater symptoms, 3 weeks after the initial brolucizumab. All patients were men and the average age was 75.0 ± 7.12 years. The patient with iridocyclitis was managed with topical steroid eye drops and the remaining two patients received subtenon triamcinolone injections. IOI was managed successfully in all patients and none had severe visual impairment. Only the patient with iridocyclitis continued PRN treatment with brolucizumab.

## Discussion

In the current study, anatomic efficacy of brolucizumab was proven in patients refractory to various types of anti-VEGF agents over a long period. As frequency of injections was once every 1.46 months, it could be assumed that fluid recurrence occurred relatively quickly. In South Korea, the injection interval for brolucizumab can be reduced to a minimum of two months, as allowed by national guidelines. One patient received brolucizumab every two months for one year (7 injections). CFT significantly decreased after switching to brolucizumab. Percentage of fluids in the different retinal compartments (SRF, PED) also decreased after switching to brolucizumab. Current treatment guidelines for nAMD suggest drying up the retina as much as possible [17]. However, some studies hypothesize a protective effect of SRF to vision threatening macular atrophy [18–20]. Similarly, some studies suggest that drying up the retina could lead to increased long-term incidence of geographic atrophy [21–23]. Regardless of the controversial fluid effect on long-term outcomes, brolucizumab was effective in drying up the retina which is the standard treatment guideline at this time.

Tachyphylaxis is a progressive decrease in therapeutic response after repeated injections of anti-VEGF agents [24]. Whenever tachyphylaxis of certain anti-VEGF agent is suspected, there is no other choice but to switch to other anti-VEGF agents. Most patients (65%) enrolled in the current study were treated with at least 2 types of anti-VEGF agents and were switched to brolucizumab because they were refractory to previous anti-VEGF agent. A substantial number of patients with tachyphylaxis were included in the study. This suggests that brolucizumab may be effective for patients who developed tachyphylaxis during repeated treatments with other anti-VEGF agents. Although which anti-VEGF agent to use in treating patients with nAMD refractory to bevacizumab/ranibizumab/aflibercept is at the individual ophthalmologist’s discretion, brolucizumab may be a valid candidate.

Unlike CFT, SFCT decreased after treatment with prior anti-VEGF agents but not after switching to brolucizumab. Although reduced pharmacologic effect on the retina has led these patients to switch therapy, the effect on the choroid had been evident with prior anti-VEGF agents. It is speculated that inhibition of VEGF leads to decreased choroidal thickness either by suppression of choroidal vascular hyperpermeability or by direct or secondary vasoconstriction induced by decreased nitric oxide production [25, 26]. Koizumi et al. demonstrate that subfoveal choroidal thickness decreased substantially during the first 3 months of monthly aflibercept injections during a 1- year study [27]. In the study, minor fluctuations with an overall plateau of choroidal thickness were formed throughout bimonthly aflibercept injections. Insignificant SFCT reduction after switching to brolucizumab in current study may be explained by two reasons; (1) ceiling effect of anti-VEGF agents on choroidal thickness and (2) 1-year follow-up period for brolucizumab which is shorter than that of prior anti-VEGF agents. CFT may have been the more accurate indicator of neovascularization reactivation than SFCT, leading to more prominent fluctuations over the course of treatment in our study.

Anatomical improvement did not translate into functional improvement. BCVA remained stable after switching to brolucizumab. Since the presence of SRF may be an indicator of viable photoreceptor and retinal pigment epithelial cells, absence of SRF after switching to brolucizumab may have led to worse BCVA in some patients [28]. Damaged photoreceptors from the long-term nAMD duration as in the patients in this study may also explain the lack of translation [29].

IOI-related adverse events which include iridocyclitis, vitritis and retinal vasculitis were observed in 7.14% of patients. This incidence is higher than that reported in the HAWK and HARRIER trials (4.6%), a real-world study performed in India (0%) and lower than the multicenter study conducted in Japan (11.3%), OCTOPUS and SWIFT trials (10.5%), and a study performed by Enríquez AB et al. (8.1%) [8, 30–33]. Prior history of IOI, female sex, and older age are some of the identified risk factors for IOI, and since different studies are comprised of variable ethnic, age, and sex groups, a direct comparison may not be possible [31, 34].

Limitations of the current study are its retrospective design, small sample size, and absence of treatment naïve patients. Retrospective design prohibited investigators to collect clinical data at more time points. Sample size may be insufficient to identify incidences of retinal vasculitis with or without occlusion which is reported to be 15.4 per 10,000 injections.

This study reports the 1-year efficacy and safety of brolucizumab in patients who were refractory to other anti-VEGF agents. Although functional improvements were not observed, anatomical improvements after numerous previous anti-VEGF agents suggest valid efficacy. Further studies regarding long term efficacy/safety in treatment of naïve patients are warranted to determine the role of brolucizumab in the current armamentarium of nAMD.
